# Neck Vibration Proprioceptive Postural Response Intact in Progressive Supranuclear Palsy unlike Idiopathic Parkinson’s Disease

**DOI:** 10.3389/fneur.2017.00689

**Published:** 2017-12-20

**Authors:** Stefan Kammermeier, Lucia Dietrich, Kathrin Maierbeck, Annika Plate, Stefan Lorenzl, Arun Singh, Kai Bötzel

**Affiliations:** ^1^Klinikum der Universität München, Neurologische Klinik und Poliklinik, München, Germany; ^2^Abteilung für Allgemeinchirurgie, Kliniken Ostallgäu-Kaufbeuren, Kaufbeuren, Germany; ^3^Klinikum der Universität München, Klinik für Anästhesiologie, München, Germany; ^4^Abteilung für Neurologie, Krankenhaus Agatharied, Hausham, Germany; ^5^Department of Neurology, University of Iowa, Iowa, IA, United States

**Keywords:** idiopathic Parkinson’s disease, progressive supranuclear palsy, posture, posturography, falling, neck vibration

## Abstract

Progressive supranuclear palsy (PSP) and late-stage idiopathic Parkinson’s disease (IPD) are neurodegenerative movement disorders resulting in different postural instability and falling symptoms. IPD falls occur usually forward in late stage, whereas PSP falls happen in early stages, mostly backward, unprovoked, and with high morbidity. Postural responses to sensory anteroposterior tilt illusion by bilateral dorsal neck vibration were probed in both groups versus healthy controls on a static recording posture platform. Three distinct anteroposterior body mass excursion peaks (P1–P3) were observed. 18 IPD subjects exhibited well-known excessive response amplitudes, whereas 21 PSP subjects’ responses remained unaltered to 22 control subjects. Neither IPD nor PSP showed response latency deficits, despite brainstem degeneration especially in PSP. The observed response patterns suggest that PSP brainstem pathology might spare the involved proprioceptive pathways and implies viability of neck vibration for possible biofeedback and augmentation therapy in PSP postural instability.

## Introduction

Idiopathic Parkinson’s disease (IPD) and the most frequent atypical Parkinsonism syndrome progressive supranuclear palsy (PSP) are neurodegenerative diseases with different postural instability features and frequent falling along the disease course.

In the alpha-synucleinopathy IPD (1–3), hypokinetic rigid motor symptoms can be well treated within the first years with dopaminergic medication. However, in advanced stage after typically 5 or more years, postural control is affected with motor freezing and falling (4), likely due to the degeneration of non-dopaminergic structures (3). Falls in this context are defined as involuntary collisions with or sliding down to a lower surface as reported by either patient or caretakers. Advanced-stage IPD patients typically fall forward, mostly while walking: by failure to initiate a walking motion, freezing, or out of a forward motion by failure to stop; body posture is in general physiological flexion with a forward shift of body mass, whereas in early stages, backward and omnidirectional falling is also observed (3, 5). Orthostatic dysfunction (6) and frontal executive disorders (3, 7) may additionally contribute to falls.

The tauopathy PSP [Steele–Richardson–Olszewski syndrome, PSP (8–11)] features typical vertical gaze disorders (12), responds poorly to dopaminergic medication and falls occur within the first year of manifestation, and frequently reported while standing or during low-velocity shifts of body mass, thus indicating at least in part deficits in adequate proprioceptive processing (13). Falls are typically unprovoked in a backward direction and without reflexive countermeasures, leading to injuries often on the back of the head with considerable morbidity (14) and even mortality. PSP falls are anamnestically related to “miniscule floor unevenness” or even no apparent reason at all.

Previous research on postural control deficits in IPD revealed inadequate sensory reweighing toward higher visual and vestibular and particularly lower proprioceptive input {as described by Vaugoyeau and Azulay (1) (platform tilt and ankle vibration), Valkovič et al. (15) [neck vibration (NV)], and Maurer et al. (13) (stabilogram diffusion analysis)} and an excessive postural correction of stance to disturbing stimuli (13, 15). Studies on postural deficits PSP are scarce [e.g., Liao et al. (16) (deficits in acoustic evoked vestibular neck reflexes), Ondo et al. (10), and Dale et al. (17) (both platform tilting)] and point toward deficits in central otolith graviception processing and limited postural boundaries of stability. The exact pathophysiological mechanisms behind PSP falls remain largely unknown. Previous PSP posture studies focused on multimodal postural challenges, usually by active platform tilting^1^ and simultaneous stimulation of all vestibular, visual, and proprioceptive systems. Indications of central sensory processing deficits largely omitted focus on the proprioceptive afferents and their adequate processing.

External NV inducing the “tonic neck reflex” is an experimental method to generate a sensory illusion in the muscle spindle receptors by simulation of passive muscle stretching [e.g., Ref. (15, 18)]. Therefore, NV appears to be a promising method to differentiate PSP and IPD proprioceptive deficits, by eliciting a whole-body multisegmental response through a proprioceptive illusion.

The aim of this study was to determine whether ambulatory PSP with typical early backward falls showed postural responses different from late-stage IPD with known pathological NV responses or healthy controls. We compared two pathophysiologically different hypokinetic rigid syndromes with clinically different modes of falling, testing whether PSP might even have a more exaggerated response than IPD, due to its brainstem degeneration. NV was used as a technically simple, readily available, and easily reproducible means to create a proprioceptive sensory illusion of whole-body multisegmental tilt.

## Materials and Methods

### Subjects

Three groups of subjects were recruited for a series of studies on static and dynamic posturography in IPD and PSP. Subject demographics and clinical scores of the individual subjects are given in Table 1A–C. All participants gave their written informed consent, and their data were anonymized at study inclusion, all in accordance with the Helsinki Declaration and to the local ethics committee (decision 142/04 of the Ethikkommission der Medizinischen Fakultät der Ludwig-Maximilians-Universität).

**Table 1:** Clinical parameters of participants in this study.

(A)				

CTR	Sex	Age	Height	Weight
1	0	58	159	54
2	1	60	173	70
3	0	51	179	73
4	1	60	163	66
5	0	60	154	60
6	0	67	168	64
7	0	57	168	63
8	1	62	180	78
9	1	46	168	105
10	0	40	167	90
11	0	73	155	57
12	0	61	168	57
13	1	60	174	81
14	0	56	159	65
15	0	60	171	88
16	0	70	165	72
17	1	69	176	100
18	1	60	185	100
19	0	60	176	81
20	1	67	180	103
21	1	42	183	95
22	1	61	183	115
Median ± SD		60 ± 8.4	170 ± 9.1	76 ± 18.1

(B)												
IPS	Sex	Age	Height	Weight	Years w/disease	UPDRS I	UPDRS II	UPDRS III	mod. III	I + II + mod. III	PIGD	H&Y
1	0	70	165	79	8							1
2	1	71	168	62		4	5	12	7	16	5	3
3	1	46	175	108	1	0	2	16	9	11	0	1
4	1	66	177	77	4	3	14	18	12	29	5	2.5
5	1	72	170	72	15	2	21	19	10	33	6	3
6	1	72	176	81	9	3	15	34	20	38	9	2.5
7	0	73	157	65	12	1	16	24	14	31	4	2
8	1	71	176	93	14	1	23	15	11	35	6	1
9	0	69	160	70	5	2	15	20	14	31	6	2.5
10	1	66	180	75	10	2	9	9	7	18	3	2
11	1	69	196	97	10	4	16	44	27	47	11	3
12	0	69	168	69	6	2	12	7	7	21	3	1.5
13	0	72	167	60	10	2	29	20	16	47	15	3
14	1	63	179	66	18	2	20	14	13	35	12	3
15	0	72	154	60	3	3	16	26	17	36	6	3
16	1	72	167	48	15	2	14	19	12	28	6	2.5
17	0	55	175	75	11	4	13	21	15	32	6	3
18	0	66	162	62	10	0	10	18	13	23	6	3
Median ± SD		70 ± 6.9	169 ± 9.9	71 ± 14.7	10 ± 4.6	2 ± 1.2	15 ± 6.5	19 ± 8.9	13 ± 5.1	31 ± 9.9	6 ± 3.6	2.5 ± 0.8

(C)																							
PSP	Sex	Age	Height	Weight	Months w/disease	UPDRS I	UPDRS II	UPDRS III	mod. III	I + II + mod.III	PIGD	H&Y	BBS	Golbe	PSP-staging	NNiPPs	l-Dopa	FAB	MMSE	PSPRS	SEADL	MADRS	Verum (1 = yes)
1	1	68	174	76		6	10	14	14	30	7	2.5	34	34	2	34	1	14	29	34	50	13	n.a.
2	1	70	172	87	24	0	13	19	15	28	7	2.5	49	22	2	23	1	14	29	26	80	10	1
3	1	60	176	65	1	4	7	11	10	21	14	3	53	23	2	25	1	16	29	27	90	15	1
4	1	68	168	82						0	5	3	33	43	2		1			43	80		n.a.
5	0	70	156	56	2	2	14	13	26	42	12	3	47	24	2	36	1	13	26	29	70	13	0
6	0	60	164	123	6	1	8	13	11	20	6	2.5	53	17	2	/	1	16	30	19	90	4	1
7	0	66	168	55	1	3	10	9	9	22	5	2.5	46	18	2	30	0	13	30	23	90	26	1
8	0	68	165	90	3	2	4		10	16	5	2.5	55	17	2	24	1	11	27				
9	0	65	162	62	4	6	12	17	16	34	6	2.5	45	27	2	34	1	13	28	30	70	24	0
10	1	71	165	63						0							1						
11	0	65	161	60	4	1	13	15	13	27	9	3	49	26	2	23	0	15	28	26	80	1	1
12	0	65	162	70	6	2	17	14	14	33	7	2.5	54	35	2	26	1	18	30	31	70	15	1
13	1	70	178	74	16	3	18		13	34	15	2.5	47	35	2	24	0	15	26				
14	1	63	183	88	36	3	14		16	33	10	2.5	29	37	2	29	1	11	29				
15	1	69	174	68	6	4	21	21	18	43	10	2.5	38	31	2	41	0	9	26	43	60	11	1
16	0	73	166	65	3	5	12		9	26	6	2.5		22	2	28	1	7	26				
17	1	70	178	82	6	2	12	13	5	19	3	2	41	24	2	34	1		28	38	70	5	0
18	0	65	168	75	2	3	13	17	18	34	12	3	49	42	2	22	1	14	29	38	70	17	0
19	0	64	162	68	22	2	16	13	13	31	9	2	42	34	2	26	1	14	30	39	80	12	0
20	1	75	170	76	1	3	8		13	24	5	2		25	2	27	1	11	27				
21	1	69	183	102	9	2	14	13	11	27	5	2	48	34	2	25	0	17	29	30	80	3	
Median ± SD		68 ± 3.9	168 ± 7.5	74 ± 16.3	5 ± 9.7	3 ± 1.6	13 ± 4.1	14 ± 3.2	13 ± 4.5	27 ± 11.1	7 ± 3.3	2.5 ± 0.3	47 ± 7.6	27 ± 7.9	2 ± 0	27 ± 5.4		14 ± 2.8	29 ± 1.5	30 ± 7.2	80 ± 11	13 ± 7.4	

*Controls subjects (CTR), idiopathic Parkinson’s disease (IPD), and progressive supranuclear palsy (PSP) are shown with sex (0 female and 1 male) (values are represented as median ± SD), age, height in centimeter, weight in kilogram, disease duration (years diagnosed with disease in IPD and months in PSP), and clinical scores Unified Parkinson Disease Rating Score (UPDRS) with items I, II, III and modified III (scaled each question/task 0–4), postural instability and gait difficulty (PIGD), and Hoehn & Yahr Scale (H&Y) along with 0–4 rated items extremity and axial rigidity; for PSP, apply specifically: Berg Balance Scale (BBS), Golbe Score, PSP staging scale, the scale of the NNiPPS study (Neuroprotection and Natural History in Parkinson Plus syndromes), frontal assessment battery (FAB), Mini-Mental State Examination (MMSE), PSP rating scale (PSPRS), Schwab & England activities of daily living (SEADL), and Montgomery-Åsberg depression rating scale (MADRS). Whether patients received rasagiline as participants in the PROSPERA study is indicated by in the column “Verum” (1 = yes). PSP patients who had levodopa in their medication regimen are indicated with “1” in the respective section; all IPD patients received levodopa. In the given collective, there was no significant effect of clinical parameters on performance in vibration effects or for Rasagiline within the PSP group*.

#### Idiopathic Parkinson’s Syndrome

Among a study pool of 20 advanced stage IPD subjects participating in a set of related studies published elsewhere (see text footnote 1),^2^ 18 were capable to participate in this study. They were 8 females/10 males ranging from 46 to 73 years of age (median, 70). They were recruited from the movement disorders outpatient clinic and selected from patients with known postural instability in the pull test and history of falls more than once a month (main inclusion criterion, anamnestically by patient and family/caretaker where applies). In the literature, clinically relevant postural instability and tendency of falling to perturbed sway have been shown repeatedly to remain at least partially resistant to medication effects [e.g., Ref. (1, 3, 13, 15)]. IPD patients still can fall regularly even under their optimal medication, and this study aimed to include them in a clinically relevant “normal everyday” state, instead of creating an artificial OFF state that does not occur in daily living conditions (3). There is also evidence that levodopa might even impair certain postural features. Therefore, patients were on their regular medication in ON state, and none had deep brain stimulation. There were no agonist-specific side effects reported. The momentary state of patients’ mobility was assessed just before the experiment with the Unified Parkinson’s Disease Rating Scale (UPDRS), Hoehn & Yahr stage, postural instability and gait difficulty scale, and the modified Schwab & England scale for recent capabilities in activities of daily living (Schwab & England activities of daily living). Rating and individual UPDRS items relevant to posture are noted in Table 1 with means and SD.

#### Progressive Supranuclear Palsy

Of 26 PSP patients clinically classified as Richardson’s syndrome subtype [ambulatory, with frequent falls as defined above, clinically probable PSP (19)], 21 were able and willing to perform the NV task (60–73 years old; median, 68; 10 females and 11 males). All were also participants of the PROSPERA study (prematurely ended, randomized double-blinded rasagiline in PSP, EudraCT number 2008-007520-26, which did not reveal an influence on disease progression). Clinical testing included (additional to those parameters also tested in IPD) PSP Rating Scale, the scale of the NNiPPS study (Neuroprotection and Natural History in Parkinson Plus syndromes, both specific PSP motor clinical scales), and neurocognitive testing due to the study medication provided such as frontal assessment battery, Mini-Mental State Examination, and Montgomery-Åsberg depression rating scale (shown with means and SD in Table 1). The extended neuropsychological testing was not performed in IPD since there was no testing for specific medication side effects and it was not in the purview of the study. Most of the PSP patients received a daily dose of levodopa (100/25 mg three to four times daily, as indicated in Table 1). They were under study medication or placebo at the time of testing, as indicated in Table 1.

#### Healthy Control Subjects

Healthy control subjects were recruited from among spouses of the patients, relatives of the authors, and former university personnel. Among a pool of 25 subjects, 22 subjects participated (age, 40–70 years; median, 60 years; 12 females and 10 males) in the study. None had history of neurological disorders of any sort or orthopedic disorders requiring surgery or regular medication.

#### Follow-up

Due to publication constraints by PROSPERA, all patients could be followed up for 4 years [compared with 0–32 months in the study by Ondo et al. (10)], in which none was re-diagnosed with a different typical or atypical Parkinsonism disorder compared to study enlistment. Also none of the control subjects developed any Parkinsonism spectrum disorder.

### Posturography

#### Experimental Setup

All subjects stood on a mechanically inert, passive recording Kistler platform with integrated piezoelectric posturography elements [9281A, Kistler Instrumente AG, Winterthur CH; e.g., used in the study by Valkovič et al. (15)]. The feet were placed together at the heels with the toes spread 30° apart. A personal computer running MATLAB 2007 (The MathWorks Inc., Natick, MA, USA, http://www.matlab.com) recorded platform signals of anteroposterior (*y*-axis), lateral (*x*-axis), and vertical (*z*) displacement of center of mass center of mass by the surrogate parameter center of foot pressure (COP) at 40 Hz together with an on/off activity signal from the vibration motor waveform generator.

#### Neck Vibration

When applied to standing subjects without additional vestibular or visual stimulation, NV generates the illusion of the whole body being flexed away multisegmentally in a pendular fashion from under the head (like the support surface slipping away) depending on the location of the vibrators: vibrating the dorsal neck bilaterally (Mm. splenius capitis, splenius cervicis) mimics swinging of the body forward and vibrating both anterior sternocleidomastoids imitates the body slipping away backward. Unilateral anterior and dorsal NV accordingly imitates a lateral slipping contralaterally. Depending on the NV mechanical impedance and frequency [50 up to 300 Hz (15, 18), and references therein], the overall compensatory postural response is a typical three-peaked body sway lasting around 2 s with the largest peak toward the side of the stimulation. According to experience from our laboratory, 80 Hz NV provides an optimal postural response (15). In addition, NV can modulate spinal reflexes, particularly H-reflex inhibition. These spinal short-latency signal integrations are likely related to the 50–100 ms range immediate postural reflex responses in comparison to the longer-latency effects in the range of 1–2 s, which are likely mediated by re-referencing of the head-centered spatial reference frame in the central vestibular system. Calf vibration, unlike NV, induces primarily ankle torsion without a multisegmental body bending and was therefore not primarily considered here since the study aimed at proprioceptive inputs simulating a whole-body displacement.

Two electromotors (Mabuchi Motor RS-385SH, Japan; 70 g weight, 0.9–14 W output, Imax = 1.06 A, 9.56 mNm maximum output, 5 V operational voltage) with an eccentric weight attached to them and each independently encased in a plastic tube (60 mm × 31 mm) with flat bottom surface (60 mm × 30 mm) previously used in the study by Valkovič et al. (15); Figure S1 in Supplementary Material were fixed over the middle of the dorsal neck paravertebrally, spaced 2 cm apart (position depicted in Figure S2 in Supplementary Material). They were firmly attached to the neck by bandages wrapped around neck and chest with loops under the arms in a horizontal figure 8, thus avoiding circular neck attachment. Direct contact with the skull was avoided (18) to minimize possible vestibular and sternocleidomastoid co-activation. They were simultaneously activated by a variable power supply and controlled by a waveform generator. When activated, pseudorandom durations of NV were applied (median, 2.50 s; actual time range, 0.92–3.27 s with preset limits 0.9–3.5 s, derived from the study by Valkovič et al. (15) and references therein) with interstimulus intervals of 5 s to counteract habituation effects. Amplitude was 1 mm at 80 Hz. The trigger delay of the device (current onset to first full revolution) in these actual devices was previously determined at 35 ms (15). The physical design and the placement of the NV motors is depicted in the Figures S1 and S2 in Supplementary Material.

### Recording Design

The posturography recording program allowed data acquisition for continuous 30-s intervals. Each individual was placed on the posturography platform with the neck vibrators in place for a total of 20 s × 30 s recording intervals. These were started simultaneously with the NV program, each followed by a brief pause. No object was closer than 1 m to the body of the subject to minimize spatial referencing. We recorded alternating eyes open (EO) and eyes closed (EC) 30-s trials with a total of 10 s × 30 s recordings for each condition (i.e., total 300 s EO and 300 s EC). Breaks up to 1 min were permitted on subject’s request after any recording. The total numbers of vibration events viable for data evaluation (inclusion: whole stimulation recorded for a total of 5 s) obtained for each group (IPD, PSP, and control) and condition (EO or EC) were a median of 25 vibrations (range, 10–38). In total for IPD, PSP, and control subjects, 437 EO and 425 EC events, 519 EO and 500 EC events, and 541 EO and 554 EC events, respectively, were eligible (variation due to the aforementioned fixed 30-s recording intervals of the static posturography system in several recording iterations). The total duration of the experiment for each subject including setup approximated 15 min.

Sudden cessation of an ongoing vibration stimulus has been shown to induce oppositely directed postural oscillations, involving multilevel spinal and supraspinal circuits (18), outside the proprioceptive purview of the study. Therefore, we focused on onset postural changes.

### Data Segmentation and Analysis

Data were segmented relative to stimulus generator onset with a 5-s segmentation window. The latency from waveform generator signal to the first full revolution of the electromotor has been previously tested to be around 35 ms in the given setup. Baseline correction and normalization were referenced to the pre-stimulus 0.5 s.

**Figure 1:**
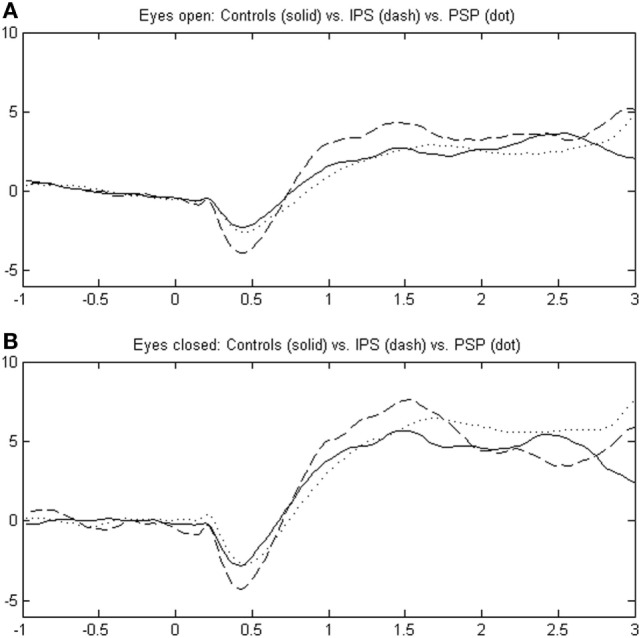
Illustrates the response pattern to neck vibration in idiopathic Parkinson’s disease (IPD), progressive supranuclear palsy (PSP), and healthy controls over a scale of 1 s before to 3 s after stimulus onset in pooled data (“grand average”); body excursions measured by center of foot pressure (COP) are scaled in centimeters in the body’s sagittal plane (anteroposterior motion) as a surrogate parameter for center of mass (COM). The upper portion **(A)** depicts the eyes open (EO) condition, and the eyes closed (EC) condition is shown in the lower graph **(B)**. Peaks are designated P1, P2, and P3 in the range of 400–1,400 ms after stimulus onset.

In all eligible stimulation events from one subject and condition (e.g., PSP5 with EO), CoP data were averaged and the resulting curve was analyzed with MATLAB for peak latencies and amplitude by least-squares curve interpolation and mathematical derivative. Three distinct postural reaction elements (15) were grouped into peaks P1–P3 (peak amplitude and latency derived through first mathematical derivative of y-axis platform signal) for further analysis with respect to, e.g., clinical scores. Figure 1 displays the averaged peaks from all subjects of one group and either visual condition.

Statistics with MATLAB and SPSS 20 used repeated measures ANOVA, Mauchley’s Sphericity/Greenhouse-Geisser correction, and Bonferroni *post hoc* correction where applies. Microsoft Excel was used for data input and transferred to aforementioned statistical applications. The level of statistical significance was set at *p* < 0.05.

## Results

### Correlation with Demographic and Clinical Scoring Data

Neither amplitude nor latency of peaks P1 through P3 correlated with demographic parameters (age or sex, even though healthy controls were significantly younger), independent of group affiliation, between-groups, or within a given group; neither applied for the EO versus EC condition. Clinical scores in the given study collectives (UPDRS for both IPD and PSP) also did not influence latencies or amplitudes of P1–P3 significantly (*p* > 0.05). It should be noted particularly that the differences in response behavior were statistically not significantly related to clinical neck rigidity (part of UPDRS rating), which is statutorily higher in PSP than in IPD. Within the PSP group, there was no statistically significant effect of study medication (rasagiline of PROSPERA) versus receiving placebo.

### General Postural Response Characteristics

Figure 1 shows the COP excursion in the anteroposterior platform plane with three distinct peaks in all three groups IPD, PSP, and controls CTR, dubbed P1, P2, and P3 [(15); EMG was used in the study by Magnusson et al. (18)]. After an initial backward motion, there was a two-peaked anterior COP excursion in all subjects.

### Group Effects

**Figure 2:**
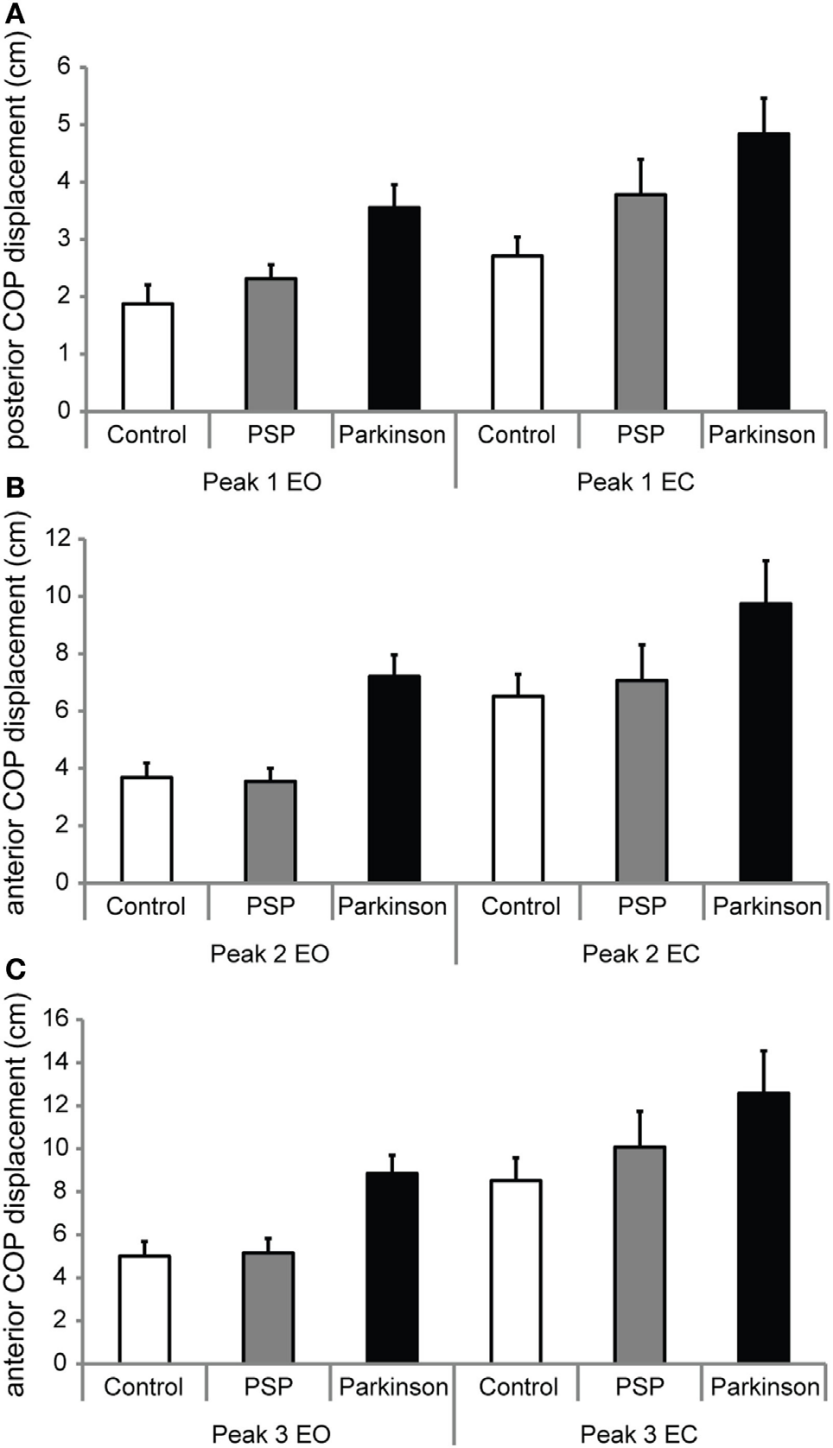
**(A,B,C)** The mean amplitudes of anteroposterior center of foot pressure (COP) displacement of peaks P1, P2, and P3, respectively, as defined in Figure 1. Amplitudes for the eyes open (EO) and eyes closed (EC) conditions are shown, which differed within-group significantly for all groups. IPD subjects exhibited larger peak amplitudes compared to control subjects across P1–P3. PSP did not exhibit differences to controls or IPD other than for P2, taking a middle ground. P2 was significantly lower for PSP than IPD. Detailed analysis is given in Table 2.

As depicted in Figure 2 and Table 2 for repeated measures ANOVA, the effects of groups (IPD, PSP, and CTR) and conditions (EO and EC) independently yielded highly significant differences for all peaks P1 through P3, but not for their relative interaction. In *post hoc* analysis of group pairs, these differences were due to significant differences in peak amplitudes between controls and IPD (larger peaks in IPD). Only for P2, IPD and PSP response amplitudes were significantly different (i.e., lower in PSP than in IPD). Comparison of peak latencies did not yield significant results between any groups.

**Table 2:** Statistical analysis of group effects with degrees of freedom for group affiliation (controls CTR, IPD, and PSP) and visual condition [eyes open (EO) or eyes closed (EC)] across peaks P1 through P3.

Peak analysis	Effect of EO versus EC Greenhouse–Geisser	Effect of group affiliation Greenhouse–Geisser	Effect of Eyes × groups interaction Greenhouse–Geisser	*Post hoc* results	
				Group pairs	*p* Value
Peak 1	*F*(1,54) = 27.19; *p* = 0.001	*F*(2,54) = 5.67; *p* = 0.006	*F*(2,54) = 0.71; *p* = 0.50	Control-IPS	*p* = 0.006
				Control-PSP	*p* = 0.378
				IPS-PSP	*p* = 0.203

Peak 2	*F*(1,54) = 26.30; *p* = 0.001	*F*(2,54) = 5.57; *p* = 0.006	*F*(2,54) = 0.26; *p* = 0.78	Control-IPS	*p* = 0.02
				Control-PSP	*p* = 0.99
				IPS-PSP	*p* = 0.04

Peak 3	*F*(1,54) = 30.93; *p* = 0.001	*F*(2,54) = 3.71; *p* = 0.03	*F*(2,54) = 0.38; *p* = 0.69	Control-IPS	*p* = 0.04
				Control-PSP	*p* = 0.89
				IPS-PSP	*p* = 0.21

*Idiopathic Parkinson’s disease (IPD) presented consistently higher peaks than controls with progressive supranuclear palsy (PSP) taking a middle ground between without significant differences to either group. Only for Peak 2, IPD amplitude was distinct from both other groups. Analysis was performed using repeated measures ANOVA with Mauchley’s Sphericity test and Greenhouse-Geisser correction*.

Considering possible effects of habituation (20), the first third of stimulation responses of each group (all events of all individuals pooled) was compared to the last third in each visual condition. There was no significant difference between first and last third for any group (PSP, IPD, and CTR) or condition (EO or EC).

## Discussion

This study studied postural responses to NV in PSP and IPD versus healthy control subjects, focused on the anteroposterior response characteristics described previously, e.g., in the study by Valkovič et al. (15). The typical three-peaked anterior postural response (15) was found in all three groups, particularly also in PSP, which had not been described previously. Its presence supports the general viability of the involved neural pathways.

In PSP, CoP amplitude responses to NV were shown to be slightly larger, but not statistically different from healthy, even younger control subjects in amplitude. This indicates that both the muscle spindle afferents and their central processing into a direct reflexive postural response remain effectively uninfluenced by PSP midbrain degeneration, even when patients show characteristic falling early in the disease course. This effect was consistent and without detectable habituation in this homogenous group of moderately affected and still ambulatory PSP patients.

Other studies in the same group of IPD and PSP published elsewhere (see text footnote 1 and text footnote 2) with an active tilting platform (simultaneous vestibular, visual, and proprioceptive stimulation) revealed primary central postural scaling deficits equivalent to exaggerated response gain for upper body segments and physical frequencies up 2 Hz. In another study with active unilateral small weight lifting, PSP showed overcompensatory postural adjustments with high-frequency oscillations in excess of the already enlarged IPD response. This NV study suggests in contrast to that overscaled sensory (particularly vestibular) input that there appears to be no exaggerated overscaling of axial proprioceptive sensor illusions.

Response latencies were no different between PSP, advanced-stage IPD, and healthy controls in contrast to the main effects observed in response amplitude. In conjunction with other studies on IPD postural disorders [e.g., Ref. (1, 2, 5, 13–15, 21, 22)], this supports the notion that primarily the spatial scaling of postural responses to defined sensory inputs is affected, instead of the signal computation and propagation of sensory information through the degenerating brainstem and basal ganglia pathways or the central scaling of responses in the temporal domain, here particularly for proprioceptive sensory stimulation. By this computational neural equivalent of higher response gain, late-stage IPD patients attempt to keep a body with reduced mechanical flexibility as close to the space vertical as possible, further away from their restricted limits of stability [in accordance with the study by Maurer et al. (13)]. The observation that IPD displayed more narrow sway characteristics than healthy controls in studies elsewhere (13) was also interpreted as an increased effort in IPD to keep COP within the narrowed limits of stability (see text footnote 1).

It may be concluded that PSP with neurodegeneration centered around the brainstem pathways important for the scaling of neck-related proprioceptive inputs might be relatively spared while patients are still ambulatory, whereas visual and particularly vestibular-related pathways are affected more intensely [(10, 16); theory of preferential degeneration]. This may be due to the anatomical localization of proprioceptive reflexes around the lower pontomedullary region in contrast to the closely intermingled oculomotor organization and vestibular processing around the midbrain (23, 24).

Alternatively, in conjunction with the typical PSP axial rigidity and restricted oculomotor capabilities due to midbrain degeneration [e.g., Ref. (8)], part of the postural compensation strategy in PSP might be a relative rescaling of axial proprioception to visual and vestibular cues. However, whether this is a cause of a deficit or a resulting compensatory strategy may be hard to differentiate. It remains to be tested whether the observed, normally scaled NV responses also apply to calf vibration stimuli, considering the normal single-joint tilt around the ankle versus a multisegmental NV response and in the light of the segment-specific response gain overscaling described elsewhere.

Practically, the normally scaled NV response in PSP might be used for physiotherapeutic and biofeedback applications [compare Ref. (25, 26)]. For example, recording multisegmental body excursions with modern 6-axes gyroscopes [compare systems proposed in Ref. (27, 28)] and counteracting excessive body motion by directed and scaled NV pulses appear as a technique to keep PSP patients mobile longer with potentially even reduced incidence of falling. Given the absence of a significant habituation effect in the large amount of stimuli >500 in a short-time course (20), NV might serve as a possible modulatory feedback tool for PSP stance stabilization, either short-term biofeedback in a physiotherapy session or for continuous use as a neural prosthetic. Further studies are warranted to study and alleviate the leading complication of the most frequent atypical Parkinson syndrome.

## Conclusion

The anteroposterior postural response of ambulatory PSP patients with typical falls to NV appears to remain intact compared to healthy controls, despite the disease-specific brainstem degeneration, unlike the known exaggerated response seen in IPD with pathophysiologically different neurodegeneration. This study indicates the basic viability of NV for feedback physiotherapy in PSP.

## Ethics Statement

This study was carried out in accordance with the recommendations of decision 142/04 of the Ethikkommission der Medizinischen Fakultät der Ludwig-Maximilians-Universität.

## Author Contributions

SK and KB composed the manuscript; SK, LD, KM, AP and AS performed the experiments; SK, AP and SL performed clinical assessment; and statistics were performed by SK and AS.

## Conflict of Interest Statement

The authors declare that the research was conducted in the absence of any commercial or financial relationships that could be construed as a potential conflict of interest.

## Footnotes

^1^Kammermeier S, Maierbeck K, Dietrich L, Plate A, Lorenzl S, Singh A, et al. Qualitative postural control differences in idiopathic Parkinson’s disease vs. progressive supranuclear palsy with dynamic-on-static platform tilt. *Clinical Neurophysiology* (2017). (under review).

^2^Kammermeier S, Dietrich L, Maierbeck K, Plate A, Lorenzl S, Singh A, et al. Postural stabilization differences in idiopathic Parkinson’s syndrome and progressive supranuclear palsy during self-triggered fast forward weight lifting. *Clinical Neurophysiology* (2017). (Under review).

## Supplementary Material

Figure S1 Image shows the custom-built neck vibration devices, each containing an electromotor (Mabuchi Motor RS-385SH, Japan; 70 g weight, 0.9–14 W output, Imax = 1.06 A, 9.56 mNm maximum output, 5 V operational voltage) with an eccentric weight attached to the axle and each independently encased in a plastic tube (60 mm × 31 mm) with flat bottom surface (60 mm × 30 mm). Setup was previously used by Valkovič et al. (14); US and EU currency displayed for size comparison.

Figure S2 Image shows the placement of the neck vibrators were fixed over the middle of the dorsal neck paravertebrally spaced 2 cm apart. They were firmly attached to the neck by bandages wrapped around neck and chest with loops under the arms in a horizontal figure 8, thus avoiding circular neck attachment. Direct contact with the skull was avoided to minimize possible vestibular and sternocleidomastoid co-activation. To demonstrate the placement of the vibrators they are shown here only partially held in place with bandage wrapping; in the actual experiment they were entirely and tightly enclosed to ensure optimal surface contact
