# Supplementary figures and images for: Neck Vibration Proprioceptive Postural Response Intact in Progressive Supranuclear Palsy unlike Idiopathic Parkinson’s Disease
Source: Front Neurol. 2017 Dec 20;8:689. doi: 10.3389/fneur.2017.00689 (PMC5742483; doi:10.3389/fneur.2017.00689)

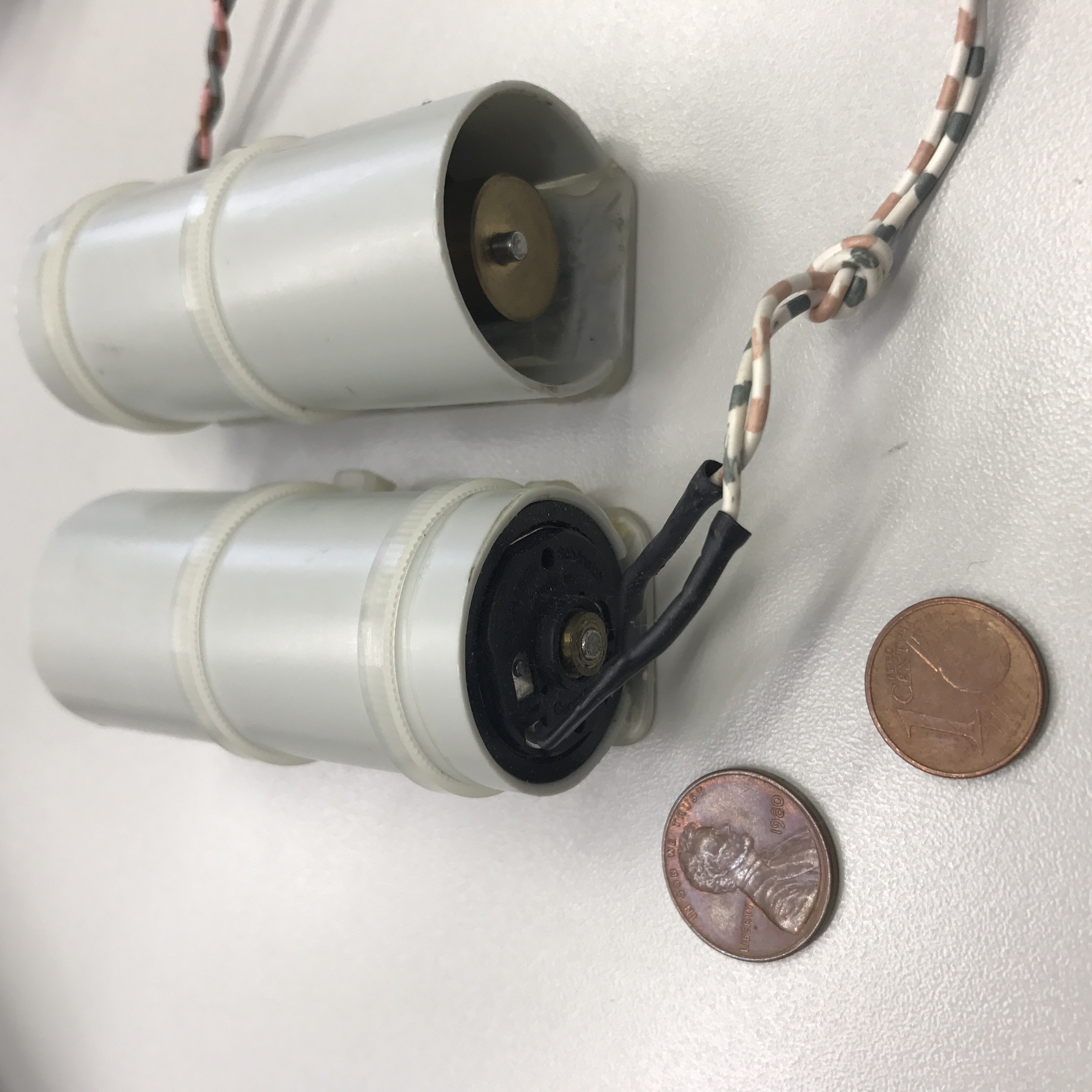

Supplement: Figure S1 — Image shows the custom-built neck vibration devices, each containing an electromotor (Mabuchi Motor RS-385SH, Japan; 70 g weight, 0.9–14 W output, Imax = 1.06 A, 9.56 mNm maximum output, 5 V operational voltage) with an eccentric weight attached to the axle and each independently encased in a plastic tube (60 mm × 31 mm) with flat bottom surface (60 mm × 30 mm). Setup was previously used by Valkovič et al. (14); US and EU currency displayed for size comparison. [file Image_1.jpeg]

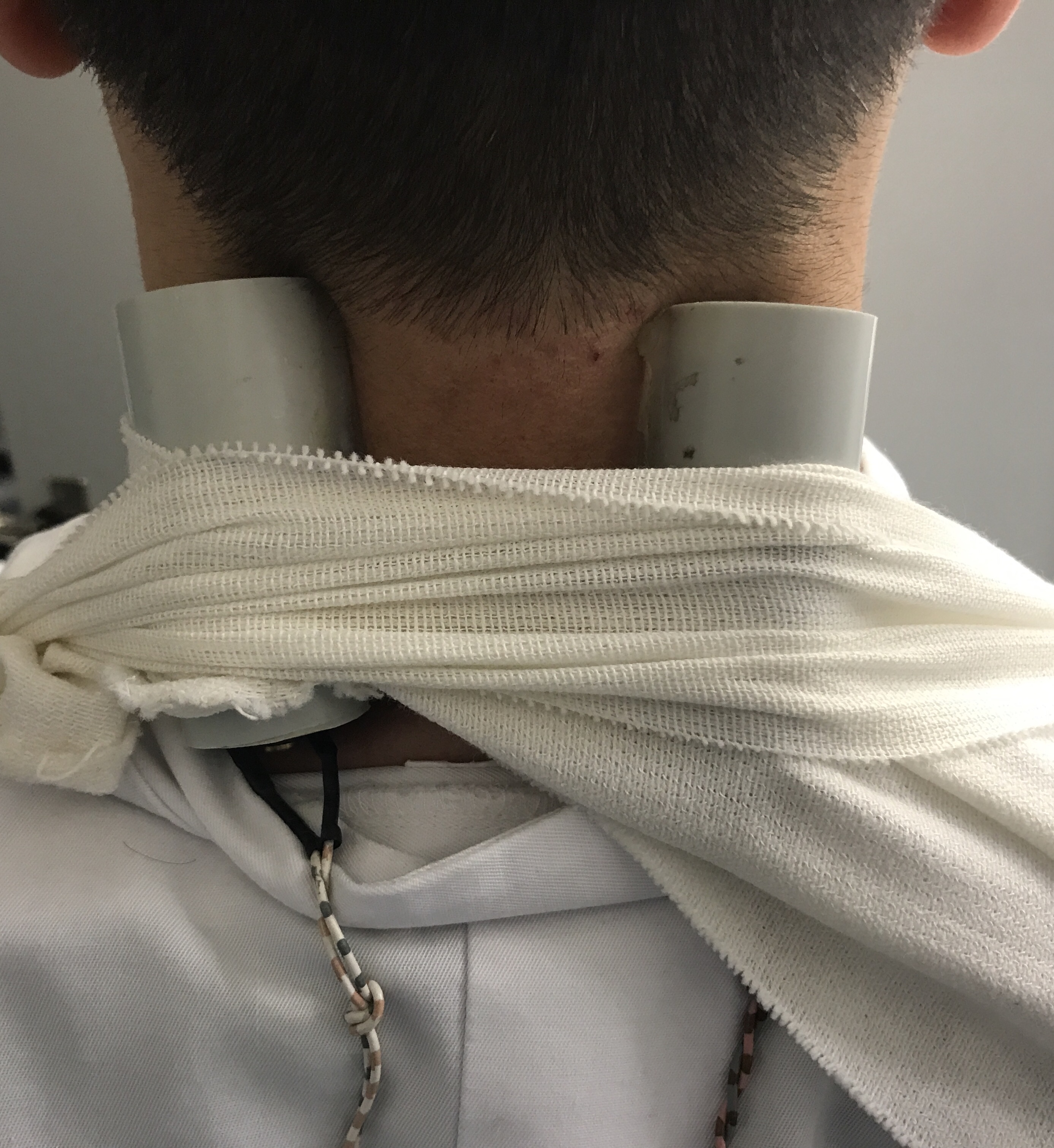

Supplement: Figure S2 — Image shows the placement of the neck vibrators were fixed over the middle of the dorsal neck paravertebrally spaced 2 cm apart. They were firmly attached to the neck by bandages wrapped around neck and chest with loops under the arms in a horizontal figure 8, thus avoiding circular neck attachment. Direct contact with the skull was avoided to minimize possible vestibular and sternocleidomastoid co-activation. To demonstrate the placement of the vibrators they are shown here only partially held in place with bandage wrapping; in the actual experiment they were entirely and tightly enclosed to ensure optimal surface contact [file Image_2.jpeg]
